# Albumin Microspheres as an Ocular Delivery System for Pilocarpine Nitrate

**DOI:** 10.4103/0250-474X.41454

**Published:** 2008

**Authors:** Sudha Rathod, S. G. Deshpande

**Affiliations:** C. U. Shah College of Pharmacy, Santacruz (W), Mumbai-400 049, India; * V. M. H. P.Shah College of Pharmacy, Ghodbunder Road, Kasarvadavli, Thane (W)-400 601, India

**Keywords:** Egg albumin, microspheres, pilocarpine nitrate, intraoccular pressure

## Abstract

Pilocarpine nitrate loaded egg albumin microspheres were prepared by thermal denaturation process in the size range of 1-12 μm. A series of batches were prepared to study factors, which may affect the size and entrapment efficiency of drug in microspheres and optimized the process. Drug loaded microspheres so obtained were evaluated for their size, entrapment efficiency, release rate and biological response. Electron photomicrographs were taken (8000X) to study the morphological characteristics of microspheres. The entrapment and encapsulation of pilocarpine after process optimization was found to be 82.63% and 62.5% respectively. In vitro dissolution rate studies revealed that the release of drug from the microspheres followed spherical matrix mechanism. Biological response of microspheric suspension was measured by reduction in intraocular pressure in albino rabbit eyes and compared with marketed eye drops. Various pharmacokinetic parameters viz. onset of action, duration of action, Tmax and AUC were studied. A measurable difference was found in the mean miotic response, duration and AUC of pilocarpine nitrate microspheric suspension.

Targeting is a controlled distribution of drug carriers in the body at specific sites. The approach utilizes carriers such as microspheres^1^, liposomes^2^, nanoparticles^3^, erythrocytes^4^, lymphocytes^5^ and macromolecules^6^ to direct the drug to its site of action. The use of natural biodegradable polymers to deliver drugs continues to be an area of active research despite the advent of synthetic biodegradable polymers.

Pilocarpine nitrate was chosen as a model drug due to ease of analysis and its effect in lowering intraocular pressure that reduces the preparation of *in vivo* models. One of the major problems of ocular therapy is to provide and maintain an adequate concentration of the drug at the site of action for a prolonged period of time. The addition of suitable polymers to liquid ophthalmic vehicles is a common method for increasing the ocular contact time and hence the drug bioavailability. Egg albumin microspheres^7^,^8^ were prepared in the size range 1-12 μm to remain undetectable by the eyes and big enough to entrap drug efficiently.

Albumin microspheres were prepared by heat stabilization process^9^. During the preparation numerous variables were found influencing the size, shape and entrapment efficiency of the microspheres^10^–^13^. The data obtained from *in vitro* release was fit into various kinetic models to study the release mechanism and release kinetics. Biological response was observed in albino rabbit eyes by measuring reduction in intraocular pressure.

## MATERIALS AND METHODS

Pilocarpine nitrate was a gift sample from JT Baker Chemicals Co., Phillisburg NJ and analyzed in the laboratory. Egg albumin was received from Loba Chemie, Mumbai, India and olive oil was purchased from Ashwin Chemicals, Mumbai, India and used as obtained. Ether and liquid paraffin were procured from SD Fine Chem Ltd., Mumbai, India, and all other chemicals and reagents used were of analytical grade.

### Preparation of microspheres:

Albumin microspheres were prepared by protein gelation process. Special apparatus was designed for the preparation of microspheres. Egg albumin was dissolved in distilled water. This solution was added drop wise in olive oil to make emulsion. From the dropping funnel, emulsion was added drop wise in the preheated olive oil (125±5°), kept in a round bottom flask, which was continuously stirred at 1500 rpm. After heat stabilization time of 10 min the preparation was cooled to 25°, centrifuged at 2500 rpm and supernatant was decanted. Microspheres thus obtained were washed with liquid paraffin and twice with ether to get a free flowing and discreet product. The microspheres were then suspended in anhydrous ether and stored at 4° in an airtight container. A number of variables were studied affecting size, shape and entrapment ability of microspheres. Separate batches were prepared and minimum of 100 particles were observed under optical microscope using oil immersion lens to optimize the variables. Observations are recorded in Table 1.

**TABLE 1:** EFFECT OF VARIOUS PROCESS VARIABLES ON THE MEAN PARTICLE SIZE OF ALBUMIN MICROSPHERES

Parameter	Mean particle size (μm)
Concentration of albumin(mg/ml)	
200	5.17
300	5.23
400	4.58
500	5.00
Stirring rate during emulsification (rpm)	
400	7.36
800	6.96
1200	4.81
Type and viscosity of oil(cps)	
Ground nut oil (169.0)	5.57
Coconut oil (130.1)	5.67
Olive oil (120.6)	4.56
Effect of drug concentration (w/v)	
5%	4.33
10%	4.58
12.5%	4.58
15.0%	5.23

Not less than 100 particles were observed using optical microscope under 100X and the mean particle size was calculated

### Electron microscopic study:

**Fig. 1:**
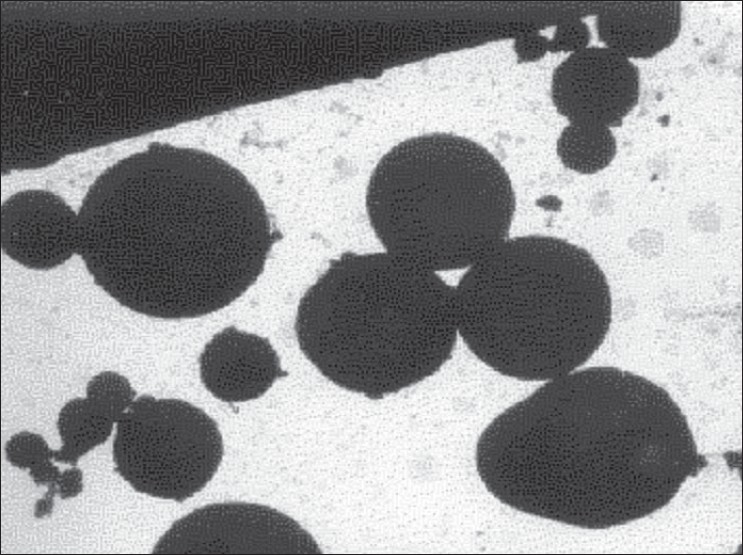
TEM photomicrograph of pilocarpine nitrate. Transmission electron microscopic photomicrograph of pilocarpine nitrate microspheres at magnification of 8000X

Photomicrographs were taken on the transmission electron microscope (model 100S, Geol Ltd., Tokyo, Japan) for the study of microspheres size and shape. Electron microscope study was carried out by placing a drop of microspheres sample on a copper grid that was dried in an oven at 60°. Copper grid was coated with phosphotungustic acid 1% w/v solution and the samples were observed under Transmission Electron Microscope. Fig. 1 Shows the photomicrograph of pilocarpine nitrate microspheres at 8,000 X.

### Optimization of process variables:

**Fig. 2:**
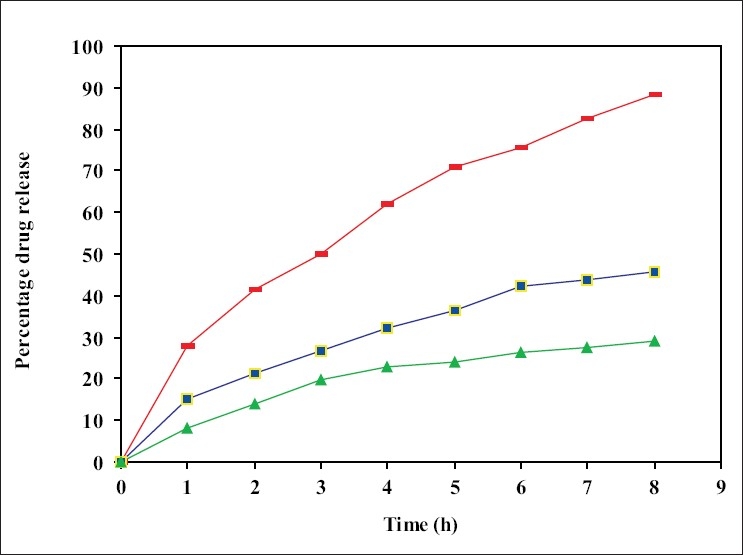
Cumulative amount of drug release from microspheres prepared at different temperature. Comparison of drug release from plain drug (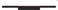), microspheres prepared at 125° (–■–) and microspheres prepared at 145° (– ▲ –).

Variables such as concentration of albumin, stirring rate during emulsification, viscosity of oil and drug concentration were studied by preparing series of batches. Results are summarized in Table 1. Other factors such as emulsion drop rate, heat stabilization temperature, stirring rate during heat stabilization of microspheres and heat stabilization time were also studied. The optimized variables are given in Table 2. Three batches of microspheres in olive oil at 105°, 125° and 145° were prepared keeping other variables same as described earlier and their release rates were studied (fig. 2).

**TABLE 2:** OPTIMUM VARIABLES IN THE PREPARATION OF DRUG LOADED ALBUMIN MICROSPHERES

Variable	Ideal condition
Albumin concentration	Aqueous solution of egg albumin 400mg/ml
Drug concentration	Aquous solution containing 50 mg/ml if pilocarpine nitrate
Rate of stirring during emulsification	1200 rpm
Emulsification time	5.0 min
Stirring rate during heat stabilization	1600 rpm
Heat stabilzation temperature	125±5°
Oil	Olive oil
Emulsion drop rate	80±5 drops per min
Heat stabilization time	10 min

Drug loaded albumin microspheres in the size range 1-12 μm were obtained by the selection of variables as above

### Analysis of surface drug:

To a portion of ether suspension of microspheres equivalents to 5 mg of PN, 0.05 ml of Tween 80 was added and the suspension was gently vortexed. Ether was then evaporated and 10 ml of 0.5 N HCl was added and centrifuged at 3000 g for 5 min. Supernatant was analyzed spectrophotometrically at 215 nm.

### Analysis of entrapped drug:

Microspheres obtained after washing were digested in 10 ml of 0.5 N HCl overnight. This solution was centrifuged to get a clear supernatant that was suitably diluted with 0.5 N HCl and assayed for PN content spectrophotometrically at 215 nm.

### Determination of *in vitro* release of PN from microspheres:

**Fig. 3:**
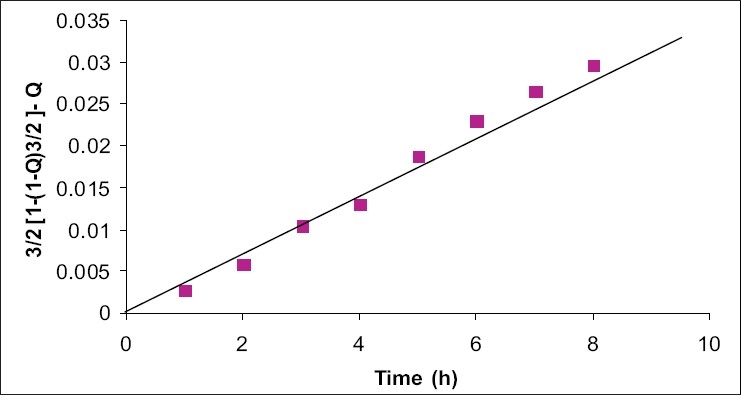
Pilocarpine nitrate release from albumin microspheres. Cumulative drug release from the optimized microsphere according to spherical matrix mechanism

Drug release was determined with the help of modified USP XXI dissolution rate model A 250 ml beaker was placed in the vessel. A plastic tube of diameter 17.5 mm opened from both the ends was tied at one end with treated cellophane membrane and dipped into the beaker containing dissolution media. Paddle type stirrer was attached in the center of the beaker and the speed was maintained at 100 rpm. The beaker was filled with 90 ml phosphate buffer (pH 7.4) and temperature was maintained at 37±1°. Albumin microspheres were suspended in 10ml of phosphate buffer. Samples were withdrawn periodically for 8 h and concentration was determined spectrophotometrically at 215 nm. Similar studies were performed taking plain drug and the data was plotted as shown in the fig. 3.

### *In vivo* studies:

*In vivo* studies were carried out in albino rabbit eyes of either sex weighing between 1.8 and 2.5 kg. All experiments were carried out at room temperature. A minimum of four rabbits were used in each experiment. Reduction in intraocular pressure was measured by Shioetz tonometer. Minimum two readings of intraocular pressure were taken prior to administration of sample. The formulation (0.05 ml) was administered with the help of an insulin syringe in the lower cul-de-sac of one eye. The control (0.05 ml) was administered in the other eye. Same animals were used repeatedly allowing minimum two days between two successive experiments. Mean reduction in intraocular pressure v/s time was plotted.

## RESULT AND DISCUSSION

Egg albumin microspheres of pilocarpine nitrate were prepared by simple emulsion technique and heat denaturation process. The microspheres obtained under these conditions were spherical, free flowing and without aggregation in the size range of 1-12 mm, which are therefore suitable for ocular administration. The effect of various process variables like albumin concentration, stirring rate, drug:albumin ratio, viscosity of oil were studied. They are summarized in Table 1. Other process variables such as heat stabilization time, stirring rate during cross linking and emulsion drop rates were found to be critical to obtain average particle size in the range 5-6 μm. Optimized variables are summarized in Table 2. Concentration of egg albumin 400 mg/ml, drug concentration 12.5% w/v, stirring rate 1200 rpm and olive oil were found to be optimum to yield desirable size of PN microspheres. Microspheres prepared at heat stabilization temperature of 105°, 125° and 145° were studied for average particle size and drug release rate. No measurable change was observed in particle size at these temperatures but a measurable change in release rate was found. Heat stabilization temp of 125° was chosen on the basis of optimum drug release from microspheres. Heat stabilization for more than 10 min resulted in charring of microspheres. Slower drop rate of 20±10 also caused charring due to prolonged heating. The entrapment and encapsulation of pilocarpine after process optimization was found to be 82.63% and 62.5% respectively. The release of active agent from the matrix involves initial swelling followed by diffusion of drug. Data obtained was treated according to the function 3/2 [1- (1- Q)^2/3^-Q] = F(t), which showed a straight line confirming the spherical matrix mechanism^14^.

Biological response of microspheric suspension was measured in albino rabbit eyes with the help of Shioetz tonometer. Pilocarpine nitrate causes reduction in intraocular pressure that can be accurately measured by Shioetz tonometer. Marketed preparations (Pilocar) 1%, 2% and 4% w/v eye drops were taken for comparative studies of 1% w/v PN microspheric suspension. Observations are recorded in Table 3. The mean miotic response was obtained as Irt = (Io – It)/Io and a plot Irt v/s time was plotted to calculate various bioavailability parameters. Observations are summerized in Table 4. Increase in concentration of PN from 1 to 4% increases the magnitude of response but not the duration of response. Marked increase in miotic response, duration and AUC of 1% PN microspheric suspension was observed as compared to the solutions.

**TABLE 3:** BIOLOGICAL RESPONSES OF (PN) EYE DROPS AND MICROSPHERES

Time (h)	Mean* IOP response of PN eye drops 1% w/v	Mean IOP response of PN eye drops 2% w/v	Mean IOP response of PN eye drops 4% w/v	Mean IOP response of PN microspheres 1% w/v
0.25	0.365±0.069	0.427±0.104	0.448±0.044	-
0.5	0.431±0.056	0.502±0.068	0.565±0.040	0.276±0.025
1.0	0.301±0.093	0.493±0.023	0.438±0.065	0.409±0.050
2.0	0.149±0.043	0.396±0.053	0.371±0.108	0.502±0.034
3.0	0.052±0.038	0.215±0.067	0.201±0.073	-
4.0	0.0	0.103±0.046	0.067±0.059	0.0369±0.030
5.0	-	0.0	0.0	-
6.0	-	-	-	0.218±0.083
8.0	-	-	-	0.122±0.081

Reduction in intraocular pressure (IOP) measured as Irt = (It - I0)/ It, It: reduction in intraocular pressure at tine t, Io : initial IOP. n = Four rabbit eyes

**TABLE 4:** MAIN BIOAVAILABILITY PARAMETERS OF SOLUTIONS AND SUSPENSIONS

Formulations	TP	Ir_t(max)_	DR	AUC
PN solution 1%	30	0.431	155	6.5
PN solution 2%	30	0.5025	240	11.6
PN solution 4%	30	0.565	225	12.45
PNM* suspension 1%	120	0.502	420	20.743

TP: Time required to achieve peak miotic response, Ir_t(max)_: Maximum miotic response calculated as (Io.It)/Io, DR: Duration of significant miotic response, AUC: Area under the curve, PNM*: pilocarpin loaded microspheres

In conclusion, these results indicate that egg albumin microspheres have potential to deliver PN for prolonged period of time. Studies can be extended to see the effect of bioadhesive gels of drug loaded microspheres for further prolongation of the effect.
